# Vitamin D Regulation of Immune Function

**DOI:** 10.1007/s11914-022-00732-z

**Published:** 2022-05-04

**Authors:** Daniel D Bikle

**Affiliations:** Veterans Affairs Medical Center, University of California San Francisco, 1700 Owens St, San Francisco, CA 94158 USA

**Keywords:** Vitamin D, Calcitriol, Innate immunity, Adaptive immunity, Pulmonary alveolar macrophage, Airway epithelia, Viral infection, Cathelicidin

## Abstract

**Purpose of Review:**

To review the mechanisms by which vitamin D and its metabolites regulate the immune system to facilitate the ability of the body to prevent and/or treat SARS-CoV2 and other respiratory infections and encourage further research into the role that vitamin D supplementation plays in preventing/treating such infections.

**Recent Findings:**

Vitamin D deficiency is associated with an increased risk of SARS-CoV2 and other respiratory infections. Clinical trials in general demonstrate that correction of vitamin D deficiency reduces the risk of hospitalization, ICU admission, and death from SARS-CoV2 infection. The airway epithelium and alveolar macrophages express the enzyme, CYP27B1, that produces the active metabolite of vitamin D, 1,25(OH)_2_D, and the vitamin D receptor, VDR. Vitamin D and its metabolites promote the innate immune response, which provides the first line of defense against viral and bacterial infections while restricting the adaptive immune response, which if unchecked promotes the inflammatory response leading to the acute respiratory distress syndrome and death.

**Summary:**

The rationale for treating vitamin D deficiency to reduce the risk of SARS-CoV2 infection and supplementing patients with vitamin D early in the course of SARS-CoV2 infection rests primarily on the ability of vitamin D metabolites to promote an effective immune response to the infection.

## Introduction

At the time of writing (November 8, 2021) SARS-CoV2, the virus causing the COVID-19 pandemic has infected over 250 million people causing more than 5 million deaths worldwide, with more each day (statistics from World Health Organization). Because testing and/or reporting is spotty in a number of countries, the likely count is much higher. The number of articles written about this virus listed in PubMed is 195,675 and climbing, a remarkable statistic given that the virus first appeared in Wuhan, China in December 2019 or less than 2 years ago. Much has been learned about the clinical and immunologic features of COVID-19 in this short period, but our understanding of mechanisms relies to a significant extent on previous epidemics/pandemics caused by other coronaviruses [1], in particular the SARS-CoV epidemic from 2002 to 2003, as well as other respiratory viruses. SARS-CoV and 2 share approximately 80% homology [2] so lessons from the immunologic consequences of the SARs-CoV infection, in both human and animal studies, provide useful insights for examining how vitamin D signaling might play a role in the current pandemic. However, differences between these viruses have been noted. Chu et al. [3•] observed in bronchial pulmonary lavage material from patients with either SARS-CoV or SARS-CoV2 that the SARS-CoV2 virus infected and replicated faster in alveolar pneumocytes and macrophages than did SARS-CoV but did not induce interferons and other proinflammatory cytokines as well, perhaps accounting for its overall greater infectivity but lower mortality.

The SARS-CoV2 virus enters the cell via its spike protein, which has two functional subunits. The S1 subunit binds the receptor, the enzyme ACE2 (angiotensin-converting enzyme 2). The other subunit, S2, promotes the fusion of the viral and host membrane leading to its internalization [4]. Epithelial cells of the upper airway have the highest concentration of ACE2, but those of the lower airway (alveolar pneumocytes) also express ACE2, albeit at lower concentrations [5•, 6]. Thus, if the infection is confined to the upper airways, the subject may remain asymptomatic, or nearly so, although infectious. Moreover, the lower expression of ACE2 in the nasal epithelium of younger children has been postulated as a reason for their decreased infection rate compared to older children and adults [7, 8]. In addition to ACE2, the serine protease TMPRSS2 also appears necessary to cleave the SARS-CoV2 protein enabling its membrane fusion and endocytosis [4]. Co-expression of both ACE2 and TMPRSS2 is found in only a small percentage of epithelial cells in both the upper and lower respiratory tract [5, 9•]. However, ACE2 also serves a protective role. It cleaves angiotensin II to angiotensin 1-7, limiting the role of the renin-angiotensin system (RAS) to suppress inflammation and fibrosis while increasing vasodilatation by binding to the MAS receptor. Similarly, ACE2 cleaves des-arg [9] bradykinin, thus removing a peptide associated with acute lung damage and inflammation [10•, 11]. Mice lacking ACE2 undergo severe lung injury [12]. SARS-CoV2 infection reduces ACE2 levels [13••], which by limiting the inhibition of both the RAS and kallikrein-kinin system contributes to the cytokine storm and recently labeled bradykinin storm [14]. On the other hand, vitamin D increases ACE2 expression while inhibiting the RAS system [15] likely contributing to its role in limiting lung injury during viral infections.

The serious damage caused by coronaviruses such as SARS-CoV and 2 is due to their infection of the lower airways with rapid virus replication, massive inflammatory cell infiltration producing a huge increase in proinflammatory cytokines and chemokines. This leads to the acute respiratory distress syndrome (ARDS), the major cause of morbidity and mortality in these infections [16]. The initial infection of the airway epithelium results in rapid viral replication [17, 18] complicated by a virus-induced delayed increase in class 1 interferon (IFNα/β) expression in dendritic cells (DC) that would normally block viral replication and enhance viral clearance by CD8 T cells [19]. The delayed expression of IFNα/β subsequently increases recruitment of proinflammatory cells, contributing to the problem. Destruction of these infected airway epithelial cells results in pyroptosis, a process of programmed cell death that leads to the secretion of a number of proinflammatory cytokines/chemokines [20]. This further dysregulates the innate immune response and attracts the influx of inflammatory cells including neutrophils, monocytes, and macrophages while sensitizing T cells to apoptosis [21, 22, 23•]. The consequences include a breakdown in the microvascular and alveolar epithelial barrier from apoptosis of the lung epithelium and endothelium resulting in vascular leakage and alveolar edema. The T cell response required for viral clearance is blunted [24, 25], and their role in dampening the cytokine storm is reduced. Recent studies using single-cell RNA-seq to define the different cell populations and what they express in bronchopulmonary lavage (BAL) specimens of patients infected with SARS-CoV2 vs other organisms found that in severe SARS-CoV2 pneumonia there was a high number of T cells thought to be recruited by infected macrophages expressing cytokines and chemokines such as CCL4 and CXCL10. These in turn expressed proinflammatory cytokines such as interferon-gamma (IFNg) that further stimulated the inflammatory macrophages [26•, 27], a vicious circle. Moreover, SARS-CoV2 infection increases the numbers of myeloid-derived suppressor cells in the lungs that could contribute to suppression of the immune response to the initial infection allowing the infection to spread [28•]. So where does vitamin D fit in? Does vitamin D with its known effects on the immune system offer benefit in either reducing the risk of infection and/or treating the infection once manifest?

## Clinical Studies Examining Vitamin D Impact on SARS-CoV2 and Other Viral Respiratory Tract Infections

The literature regarding the role of vitamin D in the prevention/management of SARS-CoV2 infections is exploding with 900 articles listed in PubMed over the past not quite 2 years. As has been pointed out by several authors, a number of host risk factors for SARS-CoV2 infections including aging, male sex, diabetes mellitus, coronary artery disease, chronic renal disease, and obesity that contribute to the severity of the infection are also associated with reduced level of 25-hydroxyvitamin D (25OHD), the primary marker of vitamin D sufficiency [29••]. Consistent with this is the negative correlation between 25OHD levels and COVID-19 positivity rate as assessed from 191,779 PCR test results [30]. Benskin [31] provides a table listing 28 systemic and basic reviews and meta-analyses specific to COVID-19 and vitamin D plus a table listing 21 intervention studies, some completed and others ongoing at the time of writing. Similarly, Teymoori-Rad and Marashi [29] provided a list of 23 trials registered with the National Institutes of Health examining the impact of vitamin D deficiency and/or supplementation on disease prevention or treatment. Although many of these are association studies [32–36, 37•, 38, 39, 40•], vitamin D supplementation appears to be protective especially in those with low levels of 25OHD at baseline and treated with modest doses at daily or weekly intervals, although the doses required may be higher than those recommended for the prevention of bone disease [39, 41•, 42]. A recent example comes from a large Spanish hospital in which 447 COVID-19-infected patients admitted to one set of wards were treated with 532mg calcifediol (25OHD) at the time of admission and 266 mg periodically throughout the next 30 days, whereas 391 patients admitted to another set of wards received standard of care without calcifediol. The assignment to which ward was random. Those treated with calcifediol had significantly fewer ICU admissions (4.5% vs 21%) and lower mortality (4.7% vs 15.9%). Initiating calcifediol after ICU admission was not effective [43]. However, the results from other randomized clinical trials (RCTs) examining the ability of vitamin D supplementation to limit viral infections have been mixed, as recently reviewed [39, 44]. Vitamin D insufficiency has also been associated with a large number of other viral infections including Epstein bar virus, varicella zoster virus, cytomegalovirus, respiratory syncytial virus, human immunodeficiency virus, hepatitis B virus, human papilloma virus, dengue [44, 45], but until randomized clinical controlled trials (RCTs) determine whether the lower levels of 25OHD are causal vs consequential (reverse causality), the role of vitamin D in these diseases remains uncertain. To this point, a Mendelian randomization study which attempted to circumvent the problem of reverse causality by evaluating whether single nucleotide polymorphisms (SNPs) associated with 25OHD levels in genome-wide association studies (GWAS) differed between patients infected with SARS-CoV2 and the general population did not find a correlation between those SNPs associated with 25OHD levels and infection rates [46]. However, these SNPs identified in GWAS studies account for only a small (approximately 5%) part of the variation in 25OHD levels. On the other hand, certain vitamin D receptor (VDR) polymorphisms have been associated with increased risk of acute lower respiratory tract infections [47].

## Regulation of the Immune Response in the Lung by Vitamin D and Its Metabolites

The respiratory tract has a large surface area (approximately 70m^2^) in contact with the environment. Thus, it provides a major site for invasion by pathogenic organisms, against which it must defend. The defense mechanism comprises both innate and adaptive immunity. Activation of the innate immune system drives activation of the long-term adaptive immune system [48]. The principal cells involved are the airway epithelia, alveolar macrophages, and dendritic cells (DC). All of these cells express CYP27B1, the enzyme producing 1,25(OH)_2_D, the active metabolite of vitamin D, and the vitamin D receptor (VDR) [49–51]. Expression of CYP27B1 is constitutive in airway epithelial cells [49], although it can be further increased by some types of viral infection [52]. The 1,25(OH)_2_D produced by these cells promotes alveolar epithelial cell proliferation and reduces their apoptosis after an inflammatory challenge [53]. Deletion of VDR from these cells leads to loss of integrity of the epithelium [54]. On the other hand, CYP27B1 is induced in alveolar macrophages by toll-like receptor (TLR) ligands for TLR1/2 (such as the lipopeptide from *mycobacterium tuberculosis*), interferonγ (IFNγ), and lipopolysaccharide (LPS) [55–57] and in DC by tumor necrosis factor alpha (TNFα), IFNγ, polyI:C, and LPS [58–60]. Moreover, these cells all express pattern recognition receptors (PRRs) of which TLRs are a major component and by which viral RNAs are recognized [61]. Neutrophils also express the VDR and can respond to 1,25(OH)_2_D, but do not appear to express CYP27B1 [62].

### Innate Immune Response

The innate immune response is initiated with the activation of PRRs in the cells of the respiratory tract. As noted above, TLRs are a major subclass of PRRs, 10 of which are functional in human cells. These TLRs detect a variety of specific membrane patterns (PAMPs) produced by various infectious agents. Bacterial ligands stimulate TLR1/2, TLR4, TLR5, and TLR2/6, whereas viral ligands stimulate TLR3, TLR7, and TLR8. Several of these TLRs utilize CD14 as a coreceptor, a molecule induced by 1,25(OH)_2_D [49]. Signaling via these TLRs includes adapter molecules such as myeloid differentiation factor 88 (MyD88) and the TIR-domain containing adapter inducing IFNb (TRIF). MyD88 signaling is increased following activation of TLRs 2, 4, 5, 7, and 9. MyD88 stimulates the translocation of NFkB into the nucleus. Indeed, one of the anti-inflammatory actions of 1,25(OH)_2_D is to block NFκB translocation to the nucleus where it would otherwise induce a panel of proinflammatory cytokines. This is achieved in part by inducing the inhibitor for NFκB translocation, IκBα that binds NFκB and keeps it in the cytoplasm [63] as will be discussed further following the discussion of adaptive immunity. This effect of 1,25(OH)_2_D does not deter its influence on viral clearance [64]. TRIF is increased following stimulation of TLR3 and 4. TRIF signaling activates interferon regulatory factor-3 (IRF-3). This molecule in turn induces type 1 interferons of which IFNα/β are the best studied. Activation of TLRs also leads to the induction of antimicrobial peptides (AMPs) and reactive oxygen species (ROS), which kill the organism. However, excess ROS can also cause cell damage, which 1,25(OH)_2_D can limit by inducing antioxidant genes such as glutathione synthase [65]. The best studied AMP produced by the activated cells is cathelicidin. The expression of this AMP is induced by 1,25(OH)_2_D in both myeloid and epithelial cells in primates but not in rodents [66, 67, 68••]. Cathelicidin has a number of actions in innate immunity. It is produced as the propeptide hCAP18 that is subsequently cleaved to its active form LL-37. Cathelicidin acts through G protein–coupled receptors to stimulate the release of IL-6 and IL-10, through ERK/P38 to increase IL-18, and through the epidermal growth factor (EGF) receptor to activate STAT1 and 3 signaling. Cathelicidin modulates TLR signaling by enhancing recognition of dsRNA, increasing binding of dsRNA binding to cell surface scavenger receptors thus increasing endocytosis, inducing the chemotaxis of neutrophils, monocytes, macrophages, and T cells into the site of infection, and promoting the clearance of respiratory pathogens by inducing apoptosis and autophagy of infected epithelial cells [45, 69]. Autophagy plays an important role in limiting the products of pyroptosis of the infected cells, thus limiting the subsequent proinflammatory response described earlier. Autophagy is promoted by vitamin D by a variety of mechanisms including inhibition of mTOR as well as stimulating cathelicidin [69, 70]. 1,25(OH)_2_D also induces another AMP, defensin β2, albeit somewhat indirectly as it involves induction of NOD2/card15/IBD1, a pattern recognition receptor for muramyl dipeptide that in turn activates NOD2 to stimulate NFκB, the direct inducer of defensin β2 [71]. Defensin β2, like cathelicidin, contributes to host defense by stimulating the expression of antiviral cytokines such as IFNβ, immune inducing molecules (NOD2, TNFα, IL-1β, IL-6), and chemokines involved in the recruitment of monocytes/macrophages, natural killer cells, neutrophils, T cells, and DC [72]. In summary, the innate immune system is the first line of defense against invading pathogens initiating the inflammatory response and activating the adaptive arm of the immune defense mechanism [73]. However, chronic activation of the innate immune response can be deleterious. 1,25(OH)_2_D inhibits TLR2, 4, 9 expression in monocytes in the later stages of activation [74, 75] and limits the excessive production of TNFα and IL-12 [76] serving to modulate chronic innate immune activity. But an additional part of the cytokine storm is due to an uncontrolled adaptive immune system, which vitamin D signaling is also poised to modulate.

### Adaptive Immune Response

The adaptive immune response involves specialized cells such as dendritic cells (DC) and macrophages that present antigen to effector cells such as T and B lymphocytes. When airway DC are activated as by a virus, they migrate to lymph nodes where they gain enhanced ability to present antigen for activation of T and B cells [77]. This activation and maturation of DC include increased CYP27B1 expression but also a decrease in VDR [59] making them less responsive to 1,25(OH)_2_D as they mature. Activation of T and B cells similarly involves priming in lymph nodes during which these cells undergo proliferation and differentiation accompanied by post translational modification of immunoglobulin production tailored to the presented antigen. Of particular relevance to this discussion is that the T helper cells vary in function. Their differentiation into the various subclasses is dependent on the context in which the antigen is presented. In general, Th1 and Th17 are proinflammatory, whereas Th2 and Th10 are anti-inflammatory. 1,25(OH)_2_D in general exerts an inhibitory action on the adaptive immune system. 1,25(OH)_2_D blocks the maturation of DC thus limiting their ability to present antigen to the effector cells [78]. Additionally 1,25(OH)_2_D inhibits the differentiation of Th1 and Th17 T cells by suppressing IL-12 production, essential for Th1 development, and IL-23 and IL-6 production essential for Th17 development. This leads to a reduction in IFNγ and IL-2 by Th1 cells and IL-17 by Th17 cells [79] further limiting recruitment of T lymphocytes (role of IFNγ), and their proliferation (role of IL-2). The decrease in IL-12 increases the development of Th2 cells which produce IL-4, IL-5, and IL-13, cytokines which shift the balance from a Th1 cell profile to a Th2 cell–dominated profile. Furthermore, 1,25(OH)_2_D increases the number of CD4+/CD25+ regulatory T cells (Treg) [80]. Treg regulate the development of the other T cell subclasses via the production of IL-10. Such regulation is involved in immune tolerance [81] and likely plays a key role in preventing the cytokine storm associated with severe respiratory disease caused by viral infections such as SARS-CoV2 [82]. On the other hand, myeloid suppressor cells that are increased in SARS-CoV infections may suppress the initial immune response to the infection, facilitating its spread. These cells, like other immune cells, express VDR, and 1,25(OH)_2_D blocks their differentiation and so their suppressive action [28].

In addition to its effects on cellular differentiation within the immune system, 1,25(OH)_2_D regulates a number of proinflammatory cytokines at the genomic and functional level. For example, TNFα has a vitamin D response element (VDRE) in its promoter through which 1,25(OH)_2_D bound to the VDR can inhibit TNFα expression. This may involve bringing an inhibitor complex perhaps containing a histone deacetylase such as HDAC3 to the promoter as has been shown for relB, an NFκB family member. Similarly, a negative VDRE has been found in the IFNγ promoter, and VDR monomers have been noted to inhibit GM-CSF expression by competing with nuclear factor of T cells 1(NFAT1) for binding to the promoter. By increasing IκBα expression, 1,25(OH)_2_D blocks the activation of NFκB and its translocation to the nucleus, which prevents genomic activation by NFκB of proinflammatory genes such as IL-8 and IL-12. As implied in the Introduction, we do not know how much of the knowledge gained about vitamin D regulated immunity in other systems applies to SARS-CoV2 infections given its recent appearance on the scene, but these studies do underlie the hope that vitamin D will be of some help in the prevention/treatment of this pandemic and provide the rationale for ongoing clinical trials in the use of vitamin D and its metabolites in the prevention/treatment of SARS-CoV2 infections.

## Conclusion

While the data clearly demonstrating a beneficial role for vitamin D in SARS-CoV2 infections are limited, its role in regulating both the innate and adaptive immune systems certainly suggests that it may be. The innate immune system is the first line of defense against invading pathogens such as viruses. It is prebuilt, relying on constitutive expression of pattern recognition receptors like TLRs to identify such pathogens. 1,25(OH)_2_D enhances that defense by inducing AMPs like cathelicidin that lead to viral destruction and clearance by several mechanisms, help recruit neutrophils, monocytes/macrophages, and DC which further the killing and clearance of these pathogens, and initiate the adaptive immune response. While beneficial acutely, chronic activation of the innate immune response is not, and can result in the cytokine/bradykinin storm. 1,25(OH)_2_D works to curtail this chronic innate immune response through a number of mechanisms including downregulation of TLRs, upregulation of ACE2, and direct inhibition of TNF/NFκB and IFNγ signaling pathways. The adaptive immune system provides a more specific response, but takes longer to develop, although once developed provides a powerful response against invading organisms. However, this response if not controlled can also be destructive. Vitamin D, via its active metabolite 1,25(OH)_2_D, regulates adaptive immunity by limiting the maturation of DC, limiting their ability to present antigen to T cells, and shifting the T cell profile from the proinflammatory Th1 and Th17 subsets to Th2 and Treg subsets, which inhibit the proinflammatory processes. Although these results come from studies with a variety of pathogens, viral and bacterial, the relevance of these protective actions on SARS-CoV2 merits further investigation.

The impact that vitamin D has on the immune system is a prime example of the broad spectrum of actions that vitamin D has on physiologic and pathologic processes. Although originally recognized as a key regulator of bone and mineral homeostasis, which it clearly is, the influence of vitamin D on the immune system illustrates its role beyond bone. That said the immune system also plays a role in the skeleton, such that although this review focuses on infection, especially by COVID-19, it is equally certain that vitamin D influences skeletal metabolism at least in part as an important constituent in osteoimmunology.
